# Taxonomic reclassification of Kaposi Sarcoma identifies disease entities with distinct immunopathogenesis

**DOI:** 10.1186/s12967-023-04130-6

**Published:** 2023-04-27

**Authors:** M. R. Openshaw, E. Gervasi, C. A. M. Fulgenzi, D. J. Pinato, A. Dalla Pria, M. Bower

**Affiliations:** 1 Institute of Cancer and Genomics Sciences, University of Birmingham, Birmingham, UK; 2 UK National Centre for HIV Oncology, Chelsea Westminster Hospital, London, UK; 3 Infectious Diseases Unit, Papa Giovanni XXIII Hospital, Bergamo, Italy; 4 Department of Surgery and Cancer, Faculty of Medicine, Hammersmith Hospital, Imperial College London, London, UK; 5 Division of Oncology, Department of Translational Medicine, University of Piemonte Orientale, Novara, Italy; 6 Department of Medical Oncology, University Campus Bio-Medico of Rome, Rome, Italy

**Keywords:** Kaposi Sarcoma, Disease classification, Human herpes virus 8, Human immunodeficiency virus, Acquired immune deficiency syndrome

## Abstract

**Background:**

The taxonomy of Kaposi Sarcoma (KS) is based on a classification system focused on the description of clinicopathological features of KS in geographically and clinically diverse populations. The classification includes classic, endemic, epidemic/HIV associated and iatrogenic KS, and KS in men who have sex with men (MSM). We assessed the medical relevance of the current classification of KS and sought clinically useful improvements in KS taxonomy.

**Methods:**

We reviewed the demographic and clinicopathological features of 676 patients with KS, who were referred to the national centre for HIV oncology at Chelsea Westminster hospital between 2000 and 2021.

**Results:**

Demographic differences between the different subtypes of KS exist as tautological findings of the current classification system. However, no definitive differences in clinicopathological, virological or immunological parameters at presentation could be demonstrated between the classic, endemic or MSM KS patients. Reclassifying patients as either immunosuppressed or non-immunosuppressed, showed that the immunosuppressed group had a significantly higher proportion of adverse disease features at presentation including visceral disease and extensive oral involvement, classified together as advanced disease (chi^2^ P = 0.0012*) and disseminated skin involvement (chi^2^ P < 0.0001*). Immunosuppressed patients had lower CD4 counts, higher CD8 counts and a trend towards higher HHV8 levels compared to non-immunosuppressed patients, however overall survival and disease specific (KS) survival was similar across groups.

**Conclusion:**

The current system of KS classification does not reflect meaningful differences in clinicopathological presentation or disease pathogenesis. Reclassification of patients based on the presence or absence of immunosuppression is a more clinically meaningful system that may influence therapeutic approaches to KS.

**Supplementary Information:**

The online version contains supplementary material available at 10.1186/s12967-023-04130-6.

## Introduction

The current categorisation of disease is largely based upon histopathology and clinical manifestations and includes several widely employed classifications such as: International Statistical Classification of Diseases and Related Health Problems (ICD9/10), Systematized Nomenclature of Medicine-Clinical Terms (SNOMED-CT), Unified Medical Language System (UMLS) and Medical Subject Headings (MeSH). These systems are based on the cataloguing of disease in the nineteenth century that originated with the biostatistician William Farr who grouped together “zymotic” or infectious diseases. The underlying principle behind these schemes was to assist in the diagnosis of diseases and perhaps help the choice of treatment. In recent times, the advances in molecular basis of disease and the associated identification of therapeutic targets for precision medicine has led to calls for a new taxonomy of disease [1, 2].

Kaposi Sarcoma (KS) is renowned in the medical and wider community as an acquired immune deficiency syndrome (AIDS) defining malignancy, causing highly visible pigmented cutaneous lesions. Those affected were highly stigmatised in the 1980s and early 1990s before the introduction of effective antiretroviral therapy. However, within the specialist medical community KS was already well known, having first been reported in 1872 by Moritz Kaposi [3]. He described KS as an aggressive pigmented sarcoma of the skin in a strikingly different group of elderly European men. Subsequently, KS has variously been subclassified into four [4] or five [5] subtypes based upon the population of people affected (Table 1).

**Table 1:** Subclassification of KS including as described by * Antman and Chang [4] and ^†^ Cesarman et al. [5]

Variant	Risk group	Typical presentation	
Classic^*†^ or sporadic	Middle aged and elderly individuals of Eastern European, Middle Eastern or Mediterranean Origin. Men affected more often than women	Lower limb cutaneous lesions. Mucosal and visceral disease are rare	Not Immunocompromised
Endemic^*†^	Individuals (children and adults) from sub-Saharan Africa, who are HIV negative	Adults present with lower limb cutaneous lesions. Children may present with lymph node involvement and lymphedema and have a more aggressive natural history	
MSM^†^	MSM without HIV infection. Often young or middle aged	Usually presents as cutaneous KS (any site). Mucosal and visceral disease are rare	
HIV associated KS^*†^	HIV infected, especially homosexual or bisexual men. KS risk rises with declining CD4 count	Multiple cutaneous lesions on limb, trunk and face. Mucosal and visceral lesions occur in 15–20% of patients	Immunocompromised
Iatrogenic^*†^	Patients receiving immunosuppressive medication. Most common in patients receiving organ transplantation	Usually presents as cutaneous KS (any site). Mucosal and visceral disease can occur	

Risk Group; population affected and Typical Presentation; most common presenting clinical features

The variety of KS that Kaposi described became known as ‘Classic’ or sporadic KS and occurs mostly in elderly and middle-aged men of Eastern European, Middle Eastern or Mediterranean origin. It typically presents as pigmented plaques or nodules on the lower limbs and frequently runs an indolent cause [6]. In the 1940s and 50s KS was described in children and adults from sub-Saharan Africa and labelled as endemic KS. In affected children the disease has a preponderance towards involvement of lymph nodes causing lymphoedema and may show more aggressive spread to the viscera, whilst in adults the disease presents in a similar manner to classic KS with plaques or nodules on the limbs, and less often invades locally or disseminates [6, 7]. In 1981 KS was described in young men who have sex with men (MSM) [8] shortly before the identification of human immunodeficiency virus (HIV) as the cause of severe immune deficiency in such patients [5]. This type of KS became known as AIDS related KS, epidemic KS or HIV associated KS and in patients with uncontrolled HIV often presents with widespread nodules and plaques on the limbs, trunk and face, and spread to organs especially the gut and lungs is not uncommon. The era of effective combination antiretroviral therapy (cART) has reduced the occurrence of KS in this groups, but even in those with well controlled HIV, cutaneous and more rarely visceral KS may occur [9]. KS has also been identified in patients who are immunocompromised due to iatrogenic causes, most commonly those seen in solid organ- or bone marrow-transplant recipients and this KS is known as iatrogenic KS [10, 11]. Finally, it has been recently recognised that MSM who are HIV negative also have an increased rate of KS, representing a newly defined fifth group, in whom KS presents in a similar manner to classic KS but in a younger age cohort [12].

The higher rate of KS in HIV infected MSM, as compared to other HIV infected populations, such as haemophiliacs [13], and the link of KS with immunosuppression were key indicators that KS had an infective cause. The causative virus, human herpesvirus-8 (HHV8), was first identified in 1994 using a technique called representational difference analysis, which isolated KS-specific DNA sequences that showed homology with known gammaherpes viruses and helped isolate the newly detected virus [14].

As the causative agent of KS, HHV8 infection is necessary to develop KS, although only a small proportion of those infected will develop KS. In addition to HHV8 infection a degree of impaired host immunity is required. This is clearly identified in HIV associated- and iatrogenic-KS, but is less well defined in the other KS variants. Theories include immunosenescence with ageing for classic KS and a combination of chronic infection and or malnutrition in endemic KS [5, 15].

The initial HHV8 infection is believed to be largely asymptomatic but similar to other herpesviruses infection is lifelong with latent and lytic phases of infection [16, 17]. KS is the most common disease caused by HHV8 infection, but the virus is also linked with primary effusion lymphoma and multicentric Castleman’s disease [17].

Given that HHV8 is required to cause KS, it is clear that the KS variants described do not have a unique pathology i.e. they are still all caused by HHV8; but rather exist on a clinico-pathological continuum caused by unique population characteristics of HHV8 prevalence, age of infection, population health and presence or absence of immune suppression. In addition, there is little evidence of significant biological differences between some of these groups in terms of clinical manifestations or histopathological features, and in many cases the therapeutic approaches are similar. We hypothesise that the most significant difference may be between KS in the immunocompetent versus KS in the immunodeficient host. To assess the medical relevance of the current classification of KS, we evaluated demographic, clinicopathological features, virological and immunological parameters at presentation, as well as overall and disease specific survival in a dataset including 676 patients all with biopsy proven KS.

## Methods

At the National Centre for HIV malignancies at the Chelsea and Westminster Hospital we prospectively collected routine data on all individuals who attend. All 676 patients diagnosed with biopsy proven Kaposi sarcoma between the years 2000 and 2021 were included in the study. Data collection was approved by the national centre for HIV malignancy. Anonymised data on patient characteristics including biological variables was extracted from the database. The date from KS diagnosis until death, study censoring or loss to follow-up was used to calculate overall survival.

Comparison of variables between the groups for categorical data was by chi^2^ test, or Fishers exact test (if one variable had a zero value), and for non-parametric continuous variables the Mann–Whitney (MW) (2 comparisons) / Kruskal–Wallis (KW) test (> 2 comparisons) were used; all P-values were two-sided. The Bonferroni correction method was used to reduce the type 1 error rate due to multiple comparisons; using the standard level of significance for a single test of P ≤ 0.05, a corrected P value of ≤ 0.0013 was required for significance (39 comparisons). Significant P-values are marked with an asterisk*. Survival was calculated from KS diagnosis until death (overall survival) or last follow-up. Survival curves were plotted according to the method of Kaplan and Meier [18]. The log rank method (Mantel-Cox proportional hazards survival analysis) was used to test for the significance of differences in survival distributions [19]. Analysis also involved disease specific (KS) survival calculated from KS diagnosis until death attributed to KS or last follow-up.

## Results

**Fig. 1:**
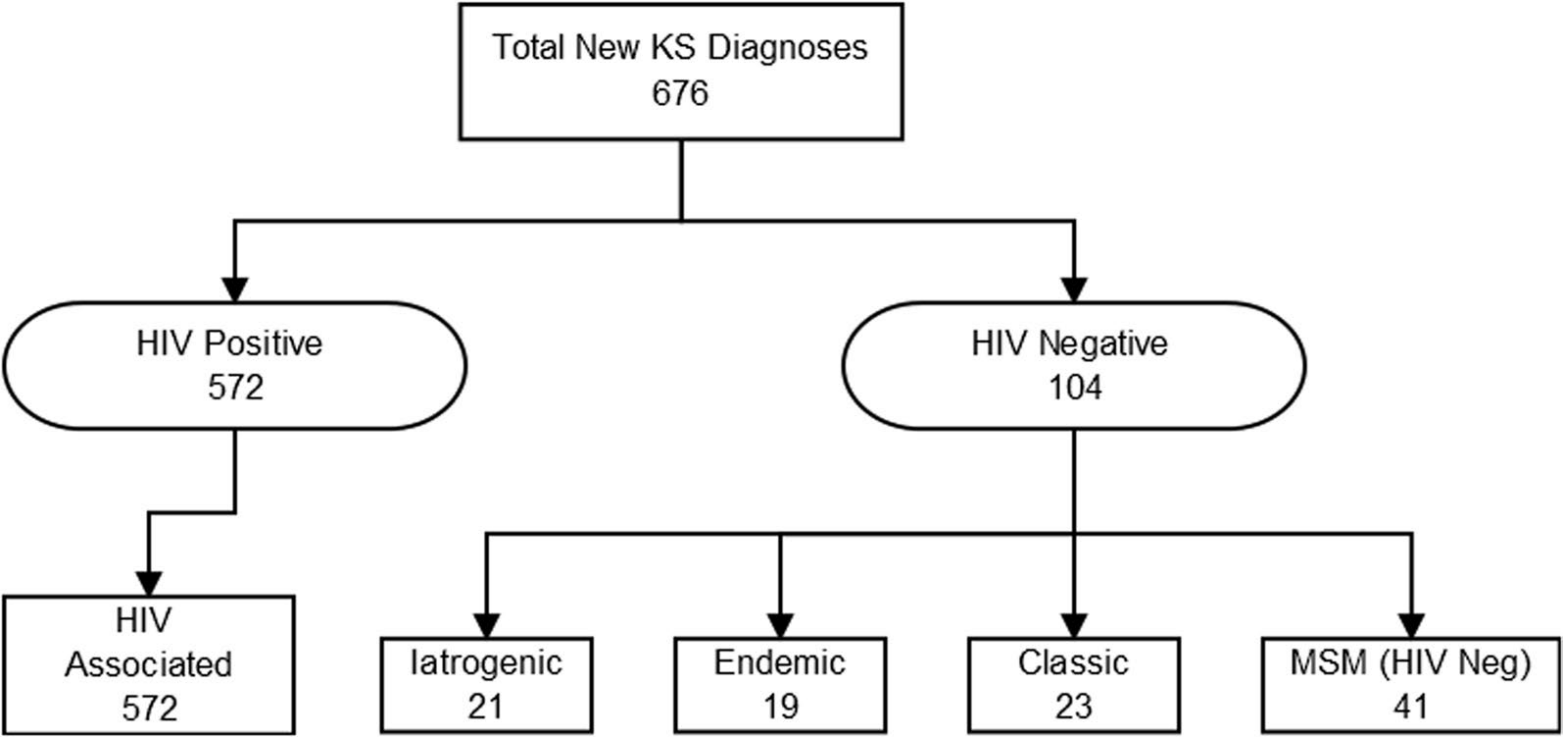
Consort diagram of patients diagnosed at Chelsea and Westminster Hospital, according to current classification system. MSM = men who have sex with men

The 676 patients were assigned at presentation into the five groups according to Cesarman, Damania [5]. The largest single group being HIV positive patients, n = 572 (HIV associated KS cohort), with the second largest grouping being MSM, n = 41 (Fig. 1) followed by classic, iatrogenic and endemic. There was a predominance of cis-gendered men who made up 92% (622/676) of the patients (Table 2). Age of presentation of KS was not evenly distributed amongst the groups, with the HIV associated mean age being youngest (41), followed by MSM (52), Iatrogenic (55) Endemic (59) and Classic (74). Overall, 98.4% of patients were first seen at our centre (and data collected) at or within 30 days of diagnosis of KS (data available for 642/676 patients). Eight patients were referred with a prior diagnosis of KS a median of 23 months (range 2–77 months) after diagnosis.

**Table 2:** Clinicopathological features of patients with KS

	HIV associated KS	Classic KS	Endemic KS	Iatrogenic KS	MSM KS	Total
Total	572	23	19	21	41	676
Gender						
*Cis*-male	531 (93%)	18 (78%)	14 (74%)	18 (86%)	41 (100%)	622 (92%)
*Cis*-female	39 (7%)	5 (22%)	5 (26%)	3 (14%)	0 (0%)	52 (7%)
*Trans*-female	2 (0.3%)	0 (0%)	0 (0%)	0 (0%)	0 (0%)	2 (1%)
Mean Age (years) Range	41 16–75	74 39–88	59 15–78	55 22–73	52 31–84	43 16–88
Race						
White-Caucasian	43 (76%)	8 (35%)	1 (5%)	6 (28%)	35 (85%)	48 (72%)
Black-African	10 (18%)	7 (30%)	13 (68%)	10 (48%)	3 (7%)	135 (20%)
Asian	32 (6%)	0 (0%)	2 (11%)	0 (0%)	2 (5%)	36 (5%)
Other	3 (0.5%)	8 (35%)	3 (16%)	5 (24%)	1 (2%)	20 (3%)
Skin sites of KS lesions						
Localised	149 (26%)	16 (69%)	10 (53%)	8 (38%)	25 (61%)	208 (31%)
Regional	73 (13%)	4 (17%)	0 (0%)	3 (14%)	1 (2%)	81 (12%)
Disseminated	324 (57%)	2 (9%)	9 (47%)	8 (38%)	13 (32%)	357 (53%)
None	26 (4%)	0 (0%)	0 (0%)	2 (9%)	2 (5%)	30 (4%)
Histological grades of KS						
Patch	91 (16%)	0 (0%)	0 (0%)	2 (10%)	3 (7%)	96 (14%)
Plaque	266 (47%)	7 (30%)	2 (11%)	3 (14%)	15 (37%)	293 (43%)
Nodular	169 (30%)	12 (51%)	16 (84%)	14 (67%)	20 (49%)	231 (34%)
Not otherwise specified	46 (8%)	4 (17%)	1 (5%)	2 (10%)	3 (7%)	56 (8%)
Disease Extent						
Disease limited to Skin	429 (75%)	21 (91%)	15 (79%)	11 (52%)	39 (95%)	515 (161%)
Advanced KS any site*	143 (25%)	2 (9%)	4 (21%)	10 (48%)	2 (5%)	161 (24%)
Sites of advanced KS involvement at diagnosis*						
Gastro-intestinal	50 (9%)	1 (4%)	2 (11%)	3 (14%)	0 (0%)	56 (8%)
Pulmonary	60 (10%)	2 (9%)	2 (11%)	6 (28%)	0 (0%)	70 (10%)
Other Viscera	94 (16%)	2 (9%)	4 (21%)	8 (38%)	2 (5%)	110 (16%)
Extensive oral involvement	81 (14%)	0 (0%)	0 (0%)	3 (14%)	0 (0%)	84 (12%)
Characteristics of advanced KS at diagnosis						
Tumour Associated Odema	87 (15%)	6 (26%)	8 (42%)	6 (28%)	3 (7%)	110 (16%)
Tumour Ulceration	60 (10%)	4 (17%)	4 (21%)	4 (19%)	3 (7%)	75 (11%)

*Sites of advanced disease defined as visceral (GI, Pulmonary, other visceral) plus extensive oral involvement

There were a number of tautological findings based on the classification system, including that the MSM group were exclusively cis-male, that the classic KS patients were older and that the endemic KS patient were more likely to be Black (Table 2). There were a number of differences in clinical presentation between the 5 subgroups, with a preponderance towards disseminated presentation of skin lesions in HIV associated KS over localised presentation, with the reverse true in classic KS and MSM KS (Table 2, Additional file 1: S1A). Nodular disease was the most common presentation for all subtypes of KS except for MSM KS. In terms of sites of advanced disease, Iatrogenic KS had more visceral, pulmonary and extensive oral involvement, with extensive oral involvement and visceral involvement also more likely with HIV associated KS. Although visceral disease was frequently found in endemic KS patients at 21% the overall numbers (n = 4) were low. Tumour oedema was least common in MSM KS, and most common in endemic KS (Table 2, Additional file 1: S1B).

**Fig. 2:**
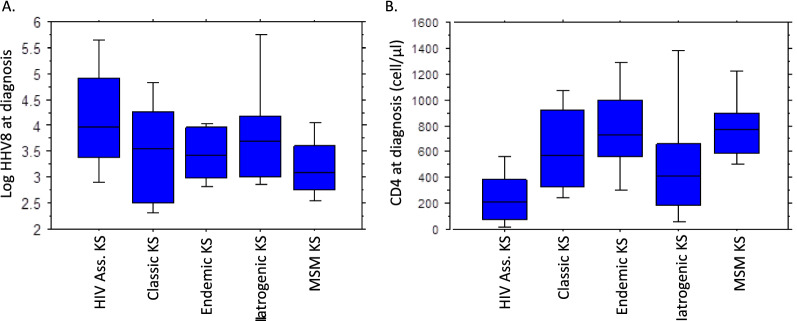
Blood parameters including HHV8 viral load and CD4 counts. **A** Box Plot of mean Log Blood HHV8 levels. **B** Box Plot of CD4 counts (cells/µl)

HHV8 bloods levels were highest in HIV associated KS followed by iatrogenic KS, and lowest in MSM KS (Table 3, Fig. 2A). CD4 levels were lowest in HIV associated KS (mean 261 cells/µl) and lower levels were also seen in iatrogenic KS (mean 543 cells/µl (Table 3, Fig. 2B)).

**Table 3:** Virological and immunological parameters according to current classification system

	HIV associated KS	Classic KS	Endemic KS	Iatrogenic KS	MSM KS	Total
Total	572	23	19	21	41	676
HHV8						
Data available for	435	15	18	16	39	523
Blood HHV8 detectable	217/435 (50%)	7/15 (47%)	9/18 (50%)	7/16 (44%)	11/39 (28%)	251/523 (48%)
Mean HHV8 viral load range (copies/ml)	1.7M 0–554M	8.1K 0–83K	2.4K 0–12K	54.6K 0–0.8M	1.1K 0–22K	1.4M 0–554M
Mean log HHV8 Viral load (range)	4.2 (1.6–8.7)	3.4 (2.2–4.9)	3.4 (2.7–4.0)	3.8 (2.8–5.9)	3.2 (2.4–4.3)	4.0 (1.6–8.7)
Leukocytes (cells/µl)						
Data available for	544	16	18	20	38	636
Mean count (range)	5.5 (1.2–24)	6.5 (4.1–8.9)	6.5 (3.8–12)	7.4 (3.4–15)	6.1 (2.9–13)	5.7 (1.2–24)
Lymphocytes (cells/µl)						
Data available for	544	16	18	17	38	633
Mean count (range)	1.7 (0.1–6.1)	1.7 (0.7–2.9)	1.8 (1.0–3.0)	1.5 (0.4–3.8)	1.7 (0.6–2.9)	1.7 (0.1–6.1)
CD4 cells/µl						
Data available for	492	12	17	15	40	576
Mean count (range)	261 (0–1850)	637 (74–1331)	788 (285–1445)	543 (39–1899)	814 (386–1779)	317(0–1850)
CD4 cell %						
Data available for	518	12	17	15	40	591
Mean count (range)	17% (0–55)	41% (11–63)	42% (19–68)	30% (5–53)	47% (33–79)	20% (0–79)
CD8 cells/µl						
Data available for	505	12	17	15	40	578
Mean count (range)	884 (0–3457)	448 (65–1099)	470 (190–855)	1214 (61–9090)	439 (172–1117)	839(0–9090)
CD8 cell %						
Data available for	503	12	17	15	40	576
Mean count (range)	59% (0–90)	27% (10–41)	27% (14–59)	39% (*16–87)	26% (12–48)	54% (0–90)

**Fig. 3:**
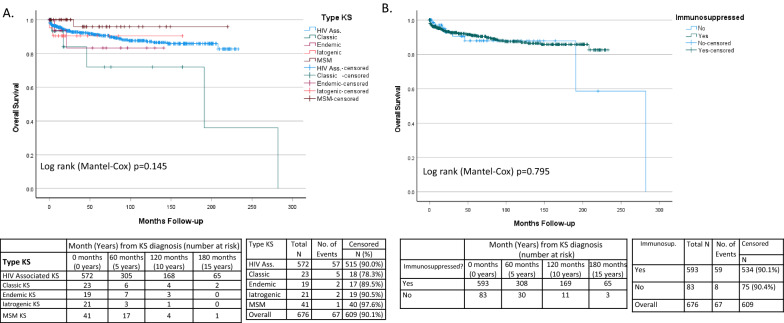
Overall survival of patients with KS. **A** Overall survival in current subtypes of KS. **B** Overall survival in immunocompromised versus non-immunocompromised patients

**Fig. 4:**
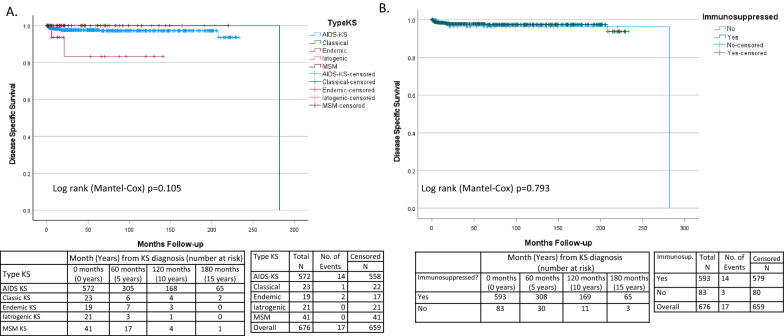
Disease Specific Survival of patients with KS. **A** Disease specific survival in subtypes of KS. **B** Disease specific survival in immunocompromised versus non-immunocompromised patients

KS was the cause of death in 17 patients, representing 25.4% of deaths. Immunosuppression related deaths (16 deaths), and AIDS defining HIV associated malignancy deaths (16 deaths) were also common (Table 4). As the majority of deaths were directly or indirectly related to immune function (KS, Immunosuppression related and AIDS defining malignancy n = 49) overall survival was the most clinically relevant endpoint. Overall survival (OS) was similar between KS subtypes (Log rank P = 0.145) and as univariate analysis was not significant, multivariant analysis was not undertaken (Fig. 3A). Overall, the number of deaths was low with median survival not reached in all apart from the classic KS subtype (Median 191 months (95% CI 0–399 months Additional file 2: S2A), which as it was more elderly had proportionally more deaths. As the median survival was only reached in a single subtype the cohort was underpowered to detect significant differences in OS (Additional file 2: S2A). Disease specific survival was also not significantly different (Log rank P = 0.105) (Fig. 4A).

**Table 4:** Cause of death of patients in the study

Cause death	Immunosuppressed	Non-immunosuppressed	N
KS	14	3	17
Immunosuppression related	16	0	16
Malignancy AIDS defining	16	0	16
Malignancy non-AIDS defining	6	1	7
Other	6	5	11
Total	58	9	67

Other includes 5 suicides

We compared classic KS to endemic KS, to determine if there were clinicopathological differences between the two classifications (Additional file 3: S3). Classic KS patients were older (mean 74 vs 59 years), but this difference is inherent within the classification system. In terms of clinicopathological differences, there were the similar levels of pulmonary (9 vs 11% chi^2^ P = 0.55), gastrointestinal (4 vs 11% chi^2^ P = 0.36), and other visceral disease involvement (9 vs 21% chi^2^ P = 0.24) and no oral involvement. There were non-significant differences in levels of tumour associated oedema (26 vs 42% chi^2^ P = 0.27) and tumour ulceration (17 vs 21% chi^2^ p = 0.76), and no difference in presenting histology (chi^2^ p = 0.09). In addition there was no difference in the proportion of patients with positive plasma HHV8 DNA (chi^2^ P = 0.71), absolute plasma HHV8 DNA levels (MW P = 0.60) or Log HHV8 levels (MW P = 0.81), and no difference in leukocyte count (MW P = 0.79), lymphocyte count (MW P = 0.91), CD4 count (MW P = 0.39), CD4% (MW P = 0.95), CD8 count (MW P = 0.51) or CD8% (MW P = 0.95) at diagnosis (Additional file 3: S3).

KS in HIV negative MSM was also reviewed and compared to classic and endemic KS. Although KS in MSM was shown to affect more white people and more men, the latter is certainly inherent of the current classification system (Table 2). MSM patients had low levels of visceral involvement with no gastrointestinal, pulmonary or oral involvement, with 5% having other visceral involvement. There was also less tumour ulceration (7%), and less tumour associated oedema (7%) in MSM. In addition, there was no difference in blood HHV8 detection (chi^2^ P p = 0.20) or mean levels (KW P = 0.128) or mean Log HHV8 (KW P = 0.660), and no significant difference in leukocyte, lymphocyte, CD4 or CD8 levels at diagnosis (Additional file 4: S4).

Given the similarities between classic and endemic KS and the low level of organ involvement and tumour ulceration or oedema in MSM KS, plus similar HHV8 and cellular levels, coupled with the observation that HIV associated and iatrogenic-KS were associated with more advanced presentation, we investigated whether an immunosuppressed (HIV associated and Iatrogenic KS) versus non-immunosuppressed (classic, endemic and MSM KS) would be a clinically suitable classification system.

**Fig. 5:**
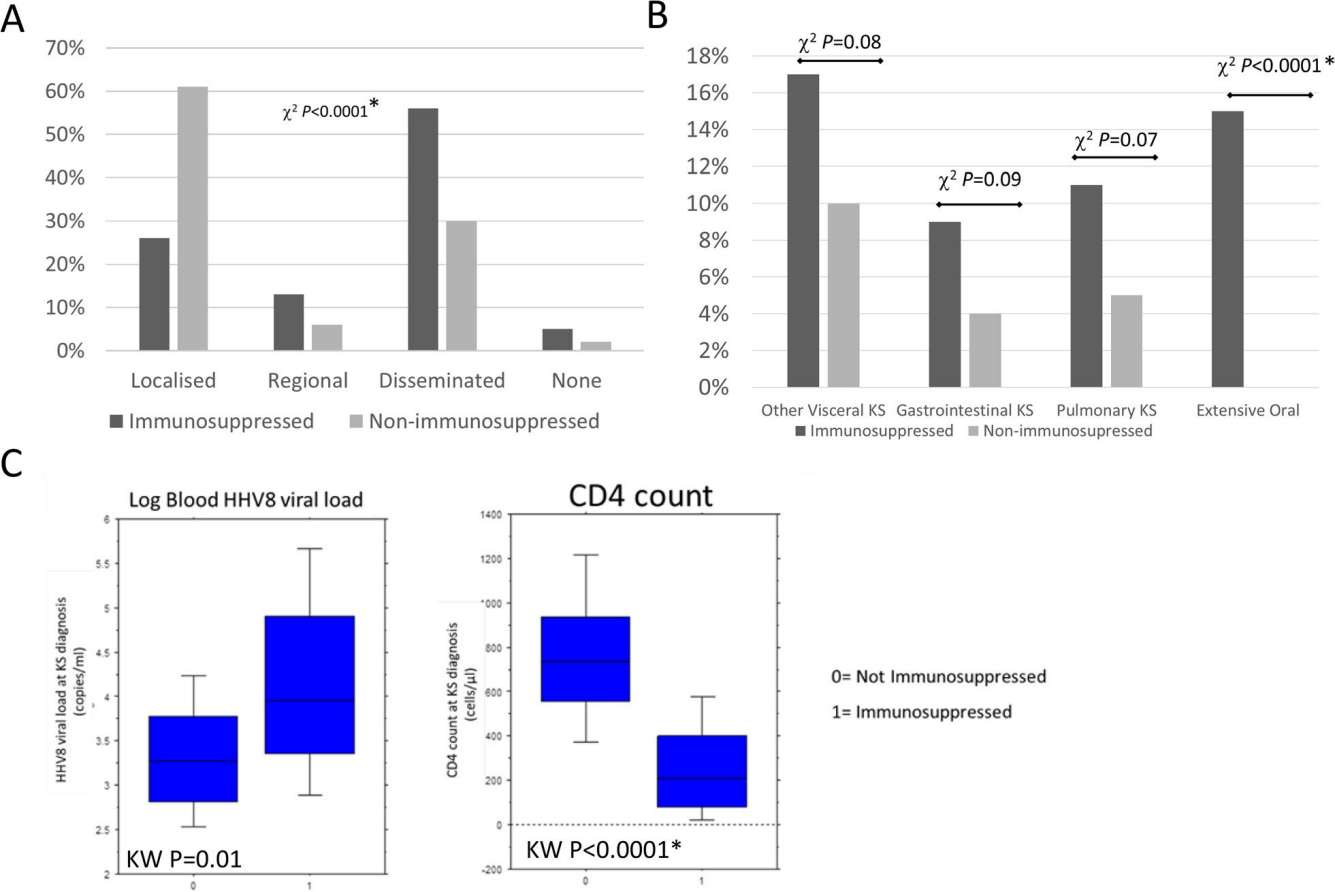
Comparison of demographic and clinicopathological characteristics, and blood parameters at presentation of KS when classified as immunosuppressed versus non-immunosuppressed. **A** Presenting Skin sites of KS lesions **B** Sites of advanced KS involvement at diagnosis. **C** Log blood HHV8 viral load and CD4 count at diagnosis

Using this classification system, the immunosuppressed group was younger (KW P < 0.0001*), whereas the non-immunosuppressed group had more Black people (chi^2^ P < 0.0001*) (Table 5). KS related deaths occurred in both groups (Table 4). Skin KS was more often disseminated (chi^2^ P < 0.0001*) and extensive oral mucosal involvement (chi^2^ P < 0.0001*) was more common in the immunosuppressed (Table 5, Fig. 5A and B). There was a trend towards more visceral involvement in the immunocompromised patients including pulmonary involvement (chi^2^ P = 0.07), gastrointestinal involvement (chi^2^ P = 0.09) and other visceral involvement (chi^2^ P = 0.08) but none reached the level of significance. Defining advanced disease as the presence of visceral disease or extensive oral involvement, showed that immunosuppressed patients were significantly more likely to have advanced disease (26% vs 8% chi^2^ = 0.0012*) compared to non-immunosuppressed patients. The immunosuppressed group had a lower leukocyte count but similar lymphocypte count, lower CD4 count (MW P < 0.001*) and CD4% (MW P < 0.001*), and higher CD8 counts (MW P < 0.001*) and CD8% (MW P < 0.001*) (Table 6). There was a trend towards higher average HHV8 viral load (MW P = 0.007) and Log HHV8 (MW P = 0.010) in the immunosuppressed group (Table 6, Fig. 5C). Overall survival was not significantly different between immunosuppressed and non-immunosuppressed (Log rank P = 0.795) (Fig. 3B). The majority of the deaths was in the immunosuppressed group, but the median survival was only reached in the non-immunosuppressed group (median 282 months (S2B)), and therefore the cohort was underpowered to detect significant differences in OS between these groups. Disease specific survival was also not significantly different (Log rank P = 0.793) (Fig. 4B).

**Table 5:** Virological and immunological parameters according to immunocompromised vs non-immunocompromised status

	Immunocompromised	Non-immunocompromised	Total	P-value
Total	593	83	676	
Gender				
*Cis*-male	549 (92.6%)	73 (88%)	622 (92%)	
*Cis*-female	42 (7.1%)	10 (12%)	52 (7%)	chi^2^ P = 0.25
*Trans*-female	2 (0.3%)	0 (0%)	2 (1%)	
Mean age (years) Range	41 16–75	74 39–88	43 16–88	MW P < 0.0001*
Race				
White-Caucasian	441 (75.5%)	44 (53%)	489 (72%)	
Black-African	112 (19%)	23 (28%)	135 (20%)	chi^2^ P < 0.0001*
Asian	32 (5%)	4 (5%)	36 (5%)	
Other	8 (0.5)	12 (14%)	20 (3%)	
Skin sites of KS lesions				
Localised	157 (26%)	51 (52%)	208 (31%)	
Regional	76 (13%)	5 (6%)	81 (12%)	chi^2^ P < 0.0001*
Disseminated	332 (56%)	25 (20%)	357 (53%)	
None	28 (5%)	2 (2%)	30 (4%)	
Histological grades of KS				
Patch	93 (16%)	3 (4%)	96 (14%)	
Plaque	269 (45%)	24 (29%)	293 (43%)	chi^2^ P < 0.0001*
Nodular	183 (31%)	48 (57%)	231 (34%)	
Not otherwise specified	48 (8%)	8 (10%)	56 (8%)	
Disease extent				chi^2^ P = 0.0012*
Disease limited to Skin	440 (74%)	75 (90%)	515 (76%)	
Advanced KS any site*	153 (26%)	8 (10%)	161 (24%)	
Sites of advanced KS involvement at diagnosis*				
Gastro-intestinal	53 (9%)	3 (4%)	56 (8%)	chi^2^ P = 0.09
Pulmonary	66 (11%)	4 (5%)	70 (10%)	chi^2^ P = 0.07
Other Viscera	102 (17%)	8 (10%)	110 (16%)	chi^2^ P = 0.08
Extensive oral involvement	84 (14%)	0 (0%)	84 (12%)	FE P < 0.0001*
Characteristics of advanced KS at diagnosis				
Tumour Associated Odema	93 (16%)	17 (20%)	110 (16%)	chi^2^ P = 0.26
Tumour Ulceration	64 (11%)	11 (13%)	75 (11%)	chi^2^ P = 0.50

^*^Sites of advanced disease defined as visceral (GI, Pulmonary, other visceral) plus extensive oral involvement

**Table 6:** Virological and immunological parameters according to immunocompromised vs non-immunocompromised status

	Immunosuppressed	Non-immunosuppressed	Total	
Total	593	83	676	
HHV8				
Data available for	451	72	523	
Blood HHV8 detectable	224/451 (49%)	27/72 (37%)	251/523 (48%)	
Mean HHV8 viral load Range (copies/ml)	1.7 M 0–554M	2.9K 0–83K	1.4M 0–554M	MW P = 0.007
Mean log HHV8 Viral load (range)	4.2(1.6–8.7)	3.3 (2.2–4.9)	4.0 (1.6–8.7)	MW P = 0.010
Leukocytes (cells/µl)				
Data available for	564	72	636	
Mean count (range)	5.6 (1.2–24)	6.4 (2.9–13)	5.7 (1.2–24)	MW P = < 0.001*
Lymphocytes (cells/µl)				
Data available for	561	72	633	
Mean count (range)	1.7(0.1–6.1)	1.7 (0.6–3.0)	1.7 (0.1–6.1)	MW P = 0.378
CD4 cells/µl				
Data available for	507	69	576	
Mean count (range)	268 (0–1899)	769 (74–1779)	317(0–1850)	MW P < 0.001*
CD4 cell %				
Data available for	533	69	591	
Mean count (range)	17% (0–55)	45% (11–79)	20% (0–79)	MW P < 0.001*
CD8 cells/µl				
Data available for	520	69	578	MW P < 0.001*
Mean count (range)	894 (0–9090)	448 (65–1117)	839(0–9090)	
CD8 cell %				
Data available for	518	69	576	MW P < 0.001*
Mean count (range)	58% (0–90)	26% (10–59)	54% (0–90)	

Exploratory analysis was completed on the duration of immunosuppression. Duration of immunosuppression was defined as the time from HIV diagnosis to KS diagnosis in HIV associated KS and the time from start of iatrogenic immunosuppression to KS in the iatrogenic group. Those in whom HIV and KS diagnosis was simultaneous were excluded because the period of immunosuppression could not be defined for these patients. Data was available for 406/593 patients with a median of 45.4 months of immunosuppression (range 1–444 months).

Data was available for 392/597 HIV associated KS patients. Median time from HIV to KS diagnosis was 30 months (range 1–184 months) for those with advanced KS involvement at diagnosis (extensive oral and/or visceral) and 51.7 months for patients without advanced KS involvement (range 1–444 months) (P < 0.0001* MW). Data on duration of iatrogenic immunosuppression was available for 14/21 patients. Those with advanced iatrogenic KS involvement had longer periods of immunosuppression (35.9 months versus 63.3), however the difference was not significant (P = 0.11).

Multicentric Castleman’s disease (MCD) which is also HHV8 associated, was recorded as a concomitant diagnosis in 32 patients with HIV associated KS. Median viral load was significantly higher at KS diagnosis 506,500 copies/ml (range 0-554 M) in patients with concomitant or prior MCD versus a median of 0 copies/ml (range 0-49 M) in patients without MCD (P < 0.0001* MW). Two other patients had MCD as a concomitant diagnosis, one with iatrogenic KS (Viral load 0 copies/ml) and one with classic KS (viral load 81,300 copies/ml), indicating that MCD occurred almost exclusively in the immunosuppressed cohort.

## Discussion

The taxonomy of KS is based on a current classification system focused on the demographics and description of KS clinicopathological features in separate populations affected by KS. This system developed as KS was found to involve each group sequentially; from classic KS (1870s), to endemic KS (1940s), iatrogenic KS (1960s), HIV associated KS (1980s) and MSM KS (2010s) [4, 5]. These descriptions took no account of the causative agent, HHV8 which was only discovered in 1994, and paid only limited acknowledgment to the presence or absence of population specific modifiers including immunosuppression.

In this review of 676 consecutive patients with KS who attended the UK national HIV oncology centre at Chelsea and Westminster hospital, we have shown that a significant proportion of the variance in population demographics between the 5 subgroups of KS are tautological findings based on the characteristics of the current classification system. For example, classic KS patients were older in a group defined as ‘elderly’, similarly, there were more Black people in the endemic KS group defined geographical as patients from Africa, there were more cis-men in the HIV associated KS group (HIV remains most common in MSM in the UK [20]) and more cis-men in the MSM KS group.

Further issues with the current classification system are highlighted as follows. Endemic and classic KS classification are both based upon the geographical location of the patient. However, as migration is possible, geographical location could be based upon the place of birth or upbringing (as most HHV8 acquisition is before the age of 20 years [21]) or based on the location the patient resides at the time of KS diagnosis. Which of these definitions is preferable is not defined in the current classification system. A further issue is that classic KS patients are defined as ‘elderly’ in the classification system, but the definition of elderly will vary upon the population mean age [22] and the age of the definer. Classic KS and to a lesser extent endemic KS are also ascribed to racial groups, but is it correct to use racial group in this definition? The differences in KS presentation maybe a reflection of HHV8 seroprevalence in different geographical populations or groups rather than a true reflection of difference caused by race as identified genetically.

In this study we investigated the current classification system by comparing demographic, clinicopathological, virological and immunological parameters in the classic and endemic KS populations. Whilst demographic differences were present e.g. classic KS patients were older (mean 74 vs 59 years) KW P = 0.0004*, this difference is inherent to the current classification system. In contrast, there were no significant clinicopathological differences between these two classifications nor were there differences in blood based immune cells or HHV8 viral levels. The same was true when classic KS and endemic KS were compared to MSM KS, though overall MSM KS appeared to have a less severe clinical presentation.

Given this lack of clinicopathological or immunological differences between classic, endemic and MSM KS, which together comprise those patients defined as non-immunosuppressed, we re-interrogated the dataset based on the presence or absence of immunosuppression as it may reflect a more meaningful difference in pathogenesis of KS than the current classification system.

By reclassifying patients to a non-immunosuppressed (classic, endemic, MSM KS) or immunosuppressed (HIV associated and iatrogenic KS) system we showed that there were meaningful differences between KS clinicopathological findings at presentation. This included more disseminated skin disease and more advanced disease (including visceral and extensive oral involvement) at presentation of KS. Within the immunocompromised group, there was a trend towards more visceral involvement of each subtype, including GI, pulmonary and other visceral involvement. In terms of blood parameters there were lower CD4 counts and leuckocyte counts, higher CD8 counts and a trend towards higher HHV8 viral load in the immunocompromised group, and MCD another HHV8 associated disease was also more common. These findings show that this classification system defines a real difference in pathogenesis between the two groups, in contrast to the current classification system. This division of KS into immunosuppressed and non-immunosuppressed also has therapeutic relevance. The approach to KS in immunosuppressed individuals is to improve the immune function either with cART to treat HIV or by reducing the anti-rejection therapy in the case of allograft recipients prior to direct anti-KS therapy. In contrast in immunocompetent individuals with KS, the treatment strategies are directed against the KS lesions directly usually using surgery, radiotherapy or chemotherapy.

Potential weaknesses to this proposed classification system exist. Firstly, there is no difference in OS or disease specific survival between the two groups despite the noted clinicopathological differences, and, as the number of deaths was low, the cohorts were underpowered for overall survival analyses. The event rate was even lower for disease specific survival, which was similarly underpowered, and no distinction on the recurrence rate of KS was possible between the two groups. This study was undertaken after the widespread use of cART which produced dramatic improvement in KS survival in the HIV associated KS group [23], and thus improvement in treatment of KS [4, 5, 24] and of HIV [25] over the last 40 years is likely responsible for the low death rates and the similarities in OS and disease specific survival.

Secondly, our data set is from the national HIV oncology centre and therefore a significant number of the patients in this study have HIV associated KS which subsequently comprises the majority of the immunosuppressed group. This may bias the analysis of the data particularly in the immunosuppressed group. However, this also reflects the rarity of iatrogenic immunosuppression coupled with a potential low level of KS in the iatrogenic group which may be less exposed to the HHV8 virus, and therefore is likely to remain a small component of immunosuppressed KS numbers.

Thirdly, it is apparent that immunocompetence does not exist as a binary element but rather as a continuum. This is reflected in patients with HIV associated KS who may have regression of their disease when they start anti-retroviral therapy [24] or when iatrogenic patients have KS regression with reduction of immunosuppressive medication [5] suggesting that even within the immunosuppressed group the degree of immunosuppression is important. It is known that when patients with HIV associated-KS who have well controlled HIV grow older they may develop new KS lesions most frequently on the lower limbs in a similar manner to classic KS patients, despite having undetectable HIV RNA levels and high CD4 cell counts [9]. Research has shown that such patients with well controlled HIV who develop KS show an increase in frequency of CD4+ and CD8+ T cells with an immunosenescent phenotype (CD57+ and CD28−) and are therefore susceptible to the same process of immunosenescence with ageing that contributes to KS in classic/non-immunocompromised KS patients [26]. Data on the control of HIV by cART in those patients with HIV associated KS that may help clarify this hypothesis of dual immunosuppression and immunosenescence was not available as part of this study, but would be an important area of future research. However, the study data did show that patients with HIV associated KS presenting with skin only KS (akin to classic KS) had a significantly longer period of immunosuppression compared to those who had advanced KS, highlighting variation even among those with HIV associated KS. One explanation for this finding is that patients with HIV who have a longer duration of cART, have less immune suppression from HIV (which is well controlled) and more immunosenescence, and therefore are less likely to have advanced KS at presentation, supporting the above hypothesis. In contrast, this trend which was not detectable in the iatrogenic group where immunosuppression is less likely to lessen with time.

However, as a broad classification, the immunosuppressed versus non-immunosuppressed is a logical division as those with HIV associated KS and iatrogenic KS have factors causing additional immunosuppression compared to those that maybe in action in the non-immunosuppressed (classic/endemic/MSM) group, including immunosenescence or more poorly understood factors such as chronic infection or malnutrition [5]. As we have shown immunosuppressed patients have a more advanced presentation it is clear these patients are more likely to require anti-tumour therapy, in addition to any intervention designed to bolster the immune system, and therefore our proposed reclassification is a meaningful way to define those who may require more aggressive treatment. Further investigation of the use of KS treatments using this new classification system as a basis will therefore form an important part of future research. The expanding role of immune-based therapies for KS [27] and the reliance of this tumour on the programmed-cell death 1 pathway as a mechanism of immune escape [28] further strengthens the rationale for a classification system centred on the host immune function.

In conclusion, the data we have presented shows that the current classification system is suboptimal, and poorly defines meaningful clinical and pathogenic differences between the subgroups. We propose that a more clinically relevant classification system for KS is to divide patients into immunocompromised and non-immunocompromised groups as this is closely linked with more advanced presentation in the immunocompromised patient and can be more helpful with guiding the need for more aggressive clinical therapeutic approaches.

## Supplementary Information

**Additional file 1**. Clinical Presentation of KS. A) Distribution of skin sites of KS. B) Characteristics of Advanced KS including sites of involvement for disseminated disease tumour oedema and tumour ulceration.

**Additional file 2**. On median overall survival.

**Additional file 3**. Clinicopathological features and virological parameters of patients with Classical versus Endemic KS.

**Additional file 4**. Virological and immunological parameters according to current classification system with Classical, Endemic and MSM KS.

## Data Availability

The datasets used and/or analysed during the current study are available from the corresponding author on reasonable request.

## Abbreviations

AIDS: Acquired immune deficiency syndrome
cART: Combination antiretroviral therapy
HHV8: Human herpesvirus-8
HIV: Human immunodeficiency virus
KS: Kaposi Sarcoma
KW: Kruskal–Wallis
MW: Mann–Whitney
MSM: Men who have sex with men
