# Supplementary material for: Taxonomic reclassification of Kaposi Sarcoma identifies disease entities with distinct immunopathogenesis
Source: J Transl Med. 2023 Apr 27;21:283. doi: 10.1186/s12967-023-04130-6 (PMC10142155; doi:10.1186/s12967-023-04130-6)
Supplement: Supplementary file 1 — Additional file 1. Clinical Presentation of KS. A) Distribution of skin sites of KS. B) Characteristics of Advanced KS including sites of involvement for disseminated disease tumour oedema and tumour ulceration. [file 12967_2023_4130_MOESM1_ESM.docx]

A.


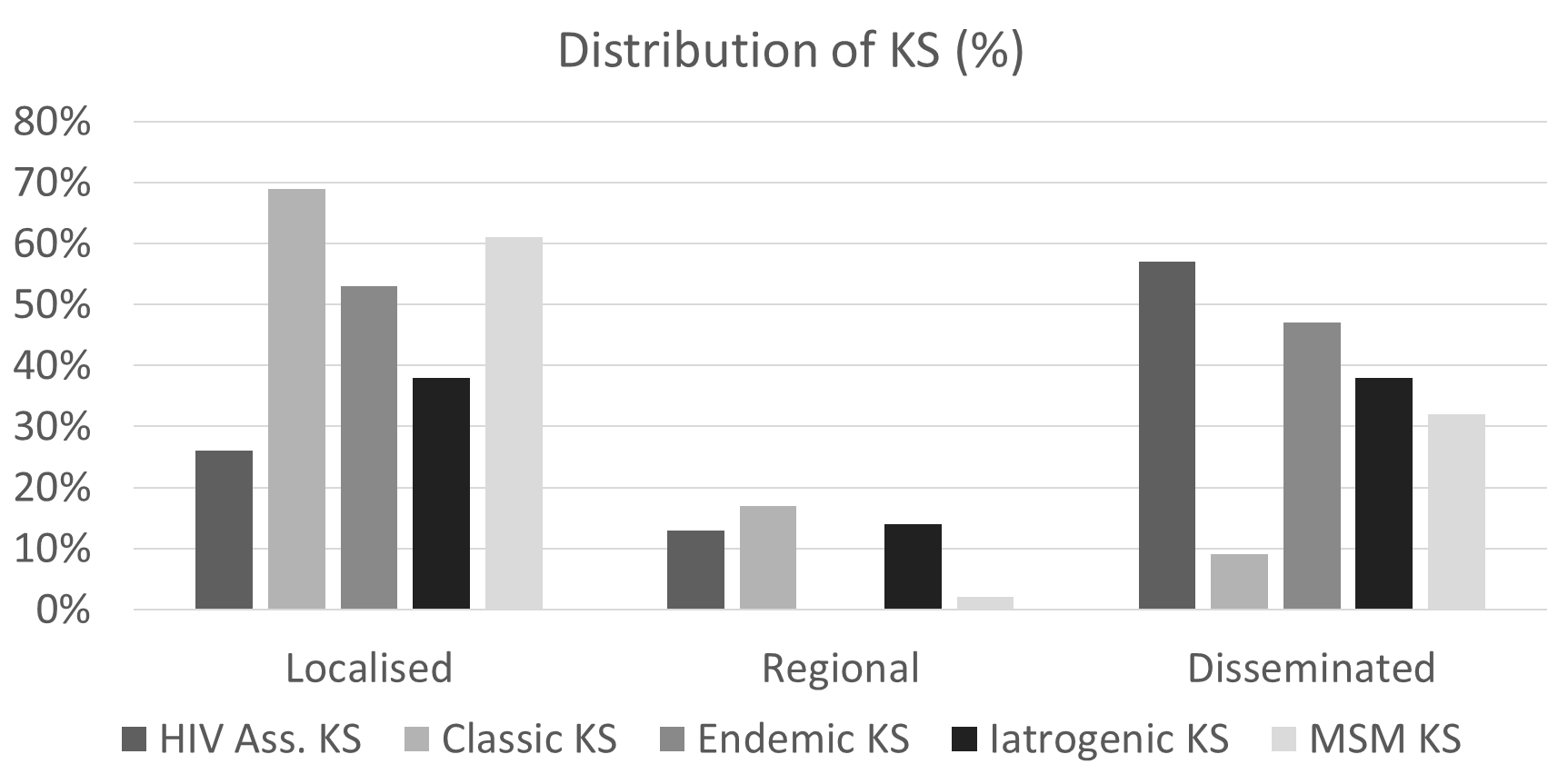


*

*

B.


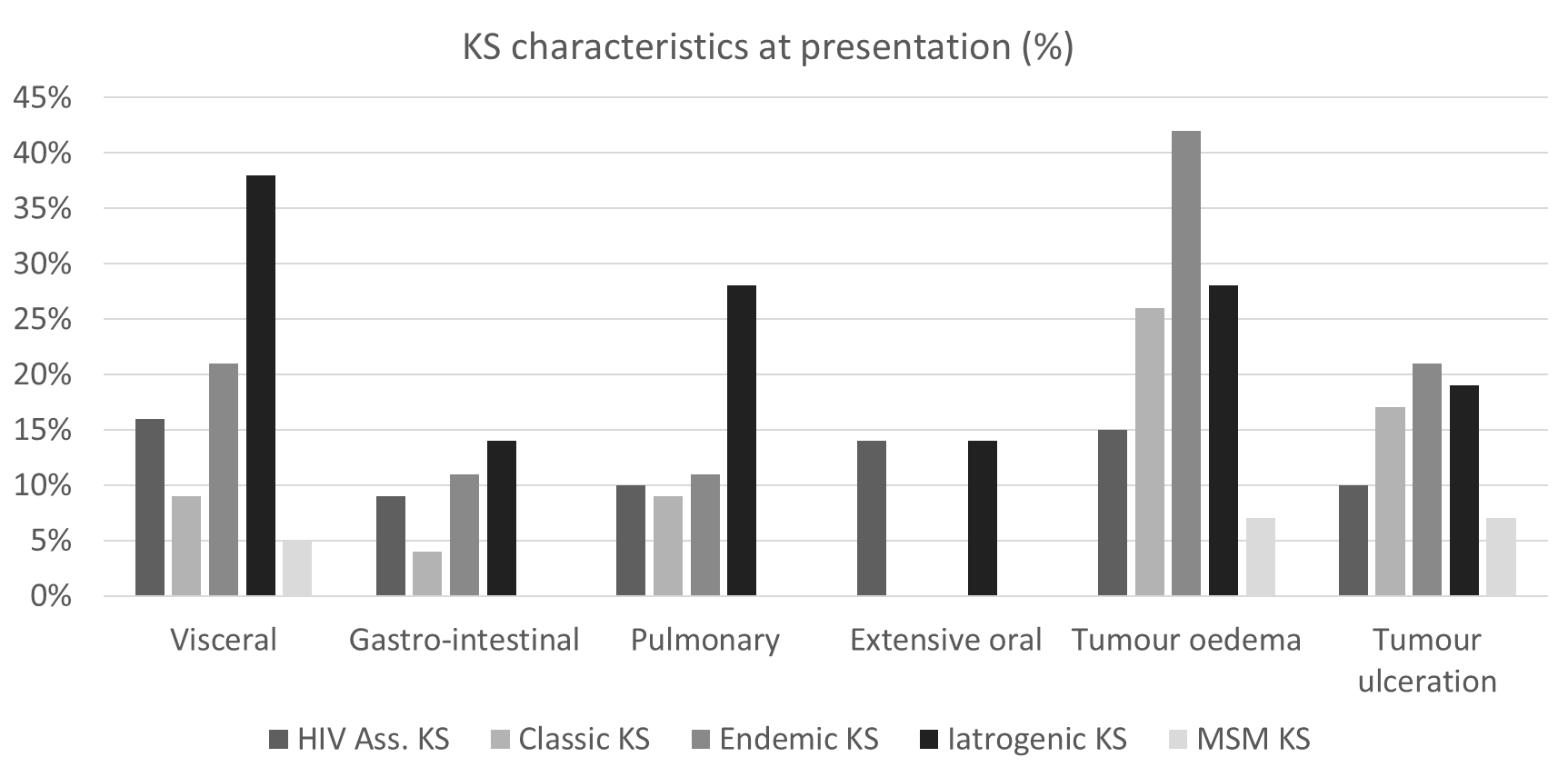


**S1 (online only). Clinical Presentation of KS.** A) Distribution of skin sites of KS. B) Characteristics of Advanced KS including sites of involvement for disseminated disease tumour oedema and tumour ulceration.
