# Supplementary material for: Taxonomic reclassification of Kaposi Sarcoma identifies disease entities with distinct immunopathogenesis
Source: J Transl Med. 2023 Apr 27;21:283. doi: 10.1186/s12967-023-04130-6 (PMC10142155; doi:10.1186/s12967-023-04130-6)
Supplement: Supplementary file 2 — Additional file 2. On median overall survival. [file 12967_2023_4130_MOESM2_ESM.docx]

**Supplementary Data 2: On Median Overall Survival**

| **S2A Means and Medians for Survival Time as per the current classification** (NR = not reached) | | | | | | | | |
| --- | --- | --- | --- | --- | --- | --- | --- | --- |
| TypeKS | Mean | | | | Median | | | |
|  | Estimate | Std. Error | 95% Conf. Interval | | Estimate | Std. Error | 95% Conf, Interval | |
|  |  |  | Lower Bound | Upper Bound |  |  | Lower Bound | Upper Bound |
| HIV Ass. | 205.94 | 3.397 | 199.28 | 212.59 | NR | . | . | . |
| Classic | 177.59 | 42.12 | 95.02 | 260.15 | 191.000 | 106.52 | .00 | 399.78 |
| Endemic | 120.06 | 13.90 | 92.83 | 147.30 | NR | . | . | . |
| Iatogenic | 148.57 | 10.48 | 128.23 | 168.90 | NR | . | . | . |
| MSM | 212.08 | 7.75 | 196.89 | 227.27 | NR | . | . | . |
|  | | | | | | | | |

| **Overall Comparisons** | | | |
| --- | --- | --- | --- |
|  | Chi-Square | df | Sig. |
| Log Rank (Mantel-Cox) | 6.826 | 4 | 0.145 |
| Test of equality of survival distributions for the different levels of TypeKS. | | | |

| **S2B Means and Medians for Survival Time for immunosuppressed vs non-immunosuppressed patients** (NR = not reached) | | | | | | | | |
| --- | --- | --- | --- | --- | --- | --- | --- | --- |
| Immunosuppressed | Mean | | | | Median | | | |
|  | Estimate | Std. Error | 95% Confidence Interval | | Estimate | Std. Error | 95% Confidence Interval | |
|  |  |  | Lower Bound | Upper Bound |  |  | Lower Bound | Upper Bound |
| No | 224.23 | 25.95 | 173.36 | 275.10 | 282.00 | .00 | . | . |
| Yes | 205.77 | 3.37 | 199.17 | 212.36 | NR | . | . | . |
| Overall | 245.28 | 4.67 | 236.13 | 254.43 | 282.0 | .00 | . | . |
|  | | | | | | | | |

| **Overall Comparisons** | | | |
| --- | --- | --- | --- |
|  | Chi-Square | df | Sig. |
| Log Rank (Mantel-Cox) | .067 | 1 | .795 |
| Test of equality of survival distributions for the different levels of Immunosuppressed. | | | |
