# Supplementary material for: Taxonomic reclassification of Kaposi Sarcoma identifies disease entities with distinct immunopathogenesis
Source: J Transl Med. 2023 Apr 27;21:283. doi: 10.1186/s12967-023-04130-6 (PMC10142155; doi:10.1186/s12967-023-04130-6)
Supplement: Supplementary file 3 — Additional file 3. Clinicopathological features and virological parameters of patients with Classical versus Endemic KS. [file 12967_2023_4130_MOESM3_ESM.docx]

|  | | Classic KS | | Endemic KS | | P-value | |
| --- | --- | --- | --- | --- | --- | --- | --- |
| Total | | 23 | | 19 | |  | |
| Gender | |  | |  | |  | |
| Cis-Men | | 18 (78%) | | 14 (74%) | |  | |
| Cis-Women | | 5 (22%) | | 5 (26%) | |  | |
| Trans-women | | 0 (0%) | | 0 (0%) | |  | |
| Race | |  | |  | |  | |
| White-Caucasian | | 8 (35%) | | 1 (5%) | | Chi ^2^ P=0.038 | |
| Black-African | | 7 (30%) | | 13 (68%) | |  |  |
| Asian | | 0 (0%) | | 2 (11%) | |  |  |
| Other | | 8 (35%) | | 3 (16%) | |  |  |
| Age at KS | |  | |  | |  | |
| Mean (range) | | 74 years (39-88) | | 59 years (15-78) | | MW P=0.0004* | |
| Skin Sites of KS lesions | |  | |  | |  | |
| Localised | | 16 (69%) | | 10 (53%) | |  | |
| Regional | | 4 (17%) | | 0 (0%) | | Chi^2^ P=0.15 | |
| Disseminated | | 2 (9%) | | 9 (47%) | |  | |
| None | | 0 (0%) | | 0 (0%) | |  | |
| Sites of advanced KS involvement at diagnosis | | | | | |  | |
| Gastro-intestinal | | 1 (4%) | | 2 (11%) | | Chi^2^ P=0.43 | |
| Pulmonary | | 2 (9%) | | 2 (11%) | | Chi ^2^ P=0.84 | |
| Other Viscera | | 2 (9%) | | 4 (21%) | | Chi ^2^ P=0.24 | |
| Extensive oral involvement | | 0 (0%) | | 0 (0%) | |  | |
| Characteristics of advanced KS at diagnosis | |  | |  | |  | |
| Tumour associated odema | | 6 (26%) | | 8 (42%) | | Chi ^2^ P=0.27 | |
| Tumour ulceration | | 4 (17%) | | 4 (21%) | | Chi ^2^ P=0.76 | |
| Histology | |  | |  | |  | |
| Patch | | 0 (0%) | | 0 (0%) | | Chi ^2^ P=0.09 | |
| Plaque | | 7 (30%) | | 2 (11%) | |  |  |
| Nodular | | 12 (51%) | | 16 (84%) | |  |  |
| Not otherwise specified | | 4 (17%) | | 1 (5%) | |  |  |
| Blood HHV8 detectable | | 7/15 (47%) | | 9/18 (50%) | | Chi ^2^ P=0.71 | |
| Mean HHV8 (Range) | | 8.1K (0-83K) | | 2.4K (0-12K) | | MW P=0.60 | |
| Mean Log KSHV (Range) | | 3.4 (2.2-4.9) | | 3.4 (2.7-4.0) | | MW P=0.81 | |
| Leukocyte count | | 6.5 (4.1-8.9) | | 6.5 (3.8-12) | | MW P=0.79 | |
| Lymphocyte count | | 1.7 (0.7-2.9) | | 1.8 (1.0-3.0) | | MW P=0.91 | |
| CD4 (cells/µl) Mean count (range) | | 637 (74-1331) | | 788 (285-1445) | | MW P=0.39 | |
| CD4 % Mean (range) | | 41% (11-63) | | 42% (19-68) | | MW P=0.95 | |
| CD8 (cells/µl) Mean count (range) | | 448 (65-1099) | | 470 (190-855) | | MW P=0.51 | |
| CD8 % Mean (range) | | 27% (10-41) | | 27% (14-59) | | MW P=0.95 | |

**S3 (online only). Clinicopathological features and virological parameters of patients with Classical versus Endemic KS.**
