# Supplementary material for: Taxonomic reclassification of Kaposi Sarcoma identifies disease entities with distinct immunopathogenesis
Source: J Transl Med. 2023 Apr 27;21:283. doi: 10.1186/s12967-023-04130-6 (PMC10142155; doi:10.1186/s12967-023-04130-6)
Supplement: Supplementary file 4 — Additional file 4. Virological and immunological parameters according to current classification system with Classical, Endemic and MSM KS. [file 12967_2023_4130_MOESM4_ESM.docx]

|  | Classic KS | Endemic KS | MSM KS | P-value |
| --- | --- | --- | --- | --- |
| Blood HHV8 detectable | 7/15 (47%) | 9/18 (50%) | 11/39 (28%) | chi^2^ P=0.20 |
| Mean HHV8 (Range) | 8.1K (0-83K) | 2.4K (0-12K) | 1.1K (0-22K) | KW P=0.128 |
| Mean Log HHV8 (Range) | 3.4 (2.2-4.9) | 3.4 (2.7-4.0) | 3.2 (2.4-4.3) | KW P=0.660 |
| Leukocyte count | 6.5 (4.1-8.9) | 6.5 (3.8-12) | 6.1 (2.9-13) | KW P=0.492 |
| Lymphocyte count | 1.7 (0.7-2.9) | 1.8 (1.0-3.0) | 1.7 (0.6-2.9) | KW P=0.973 |
| CD4 (cells/µl) Mean count (range) | 637 (74-1331) | 788 (285-1445) | 814 (386-1779) | KW P=0.305 |
| CD4 % Mean (range) | 41% (11-63) | 42% (19-68) | 47% (33-79) | KW P=0.337 |
| CD8 (cells/µl) Mean count (range) | 448 (65-1099) | 470 (190-855) | 439 (172-1117) | KW P=0.717 |
| CD8 % Mean (range) | 27% (10-41) | 27% (14-59) | 26% (12-48) | KW P=0.903 |

**S4 (online only) Virological and immunological parameters according to current classification system with Classical, Endemic and MSM KS.**
